# Pioglitazone protects blood vessels through inhibition of the apelin signaling pathway by promoting KLF4 expression in rat models of T2DM

**DOI:** 10.1042/BSR20190317

**Published:** 2019-12-24

**Authors:** Ying Wang, Ruonan Zhang, Hailin Shen, Jing Kong, Xinrui Lv

**Affiliations:** 1 The First Affiliated Hospital, Henan University, Kaifeng, Henan 475004, China; 2 The Key Laboratory of Receptors-Mediated Gene Regulation and Drug Discovery of School of Basic Medicine, Henan University, Kaifeng, Henan 475004, China; 3 School of Chinese Integrative Medicine, Hebei Medical University, Shijiazhuang 050017, China

**Keywords:** Apelin, KLF4, Macroangiopathy, Pioglitazone, STZ, VSMCs

## Abstract

Apelin, identified as the endogenous ligand of APJ, exerts various cardiovascular effects. However, the molecular mechanism underlying the regulation of apelin expression in vascular cells is poorly described. Pioglitazone (PIO) and Krüppel-like factor 4 (KLF4) exhibit specific biological functions on vascular physiology and pathophysiology by regulating differentiation- and proliferation-related genes. The present study aimed to investigate the roles of PIO and KLF4 in the transcriptional regulation of apelin in a high-fat diet/streptozotocin rat model of diabetes and in PIO-stimulated vascular smooth muscle cells (VSMCs). Immunohistochemistry, qRT-PCR, and Western blotting assays revealed that the aorta of the Type 2 diabetes mellitus (T2DM) rat models had a high expression of apelin, PIO could decrease the expression of apelin in the PIO-treated rats. *In vitro*, Western blotting assays and immunofluorescent staining results showed that the basal expression of apelin was decreased but that of KLF4 was increased when VSMCs were stimulated by PIO treatment. Luciferase and chromatin immunoprecipitation assay results suggested that KLF4 bound to the GKLF-binding site of the apelin promoter and negatively regulated the transcription activity of apelin in VSMCs under PIO stimulation. Furthermore, qRT-PCR and Western blotting assay results showed that the overexpression of KLF4 markedly decreased the basal expression of apelin, but the knockdown of KLF4 restored the PIO-induced expression of apelin. In conclusion, PIO inhibited the expression of apelin in T2DM rat models to prevent diabetic macroangiopathy, and negatively regulated the gene transcription of apelin by promoting transcription of KLF4 in the apelin promoter.

## Introduction

The incidence of Type 2 diabetes mellitus (T2DM) is currently very high, with several young individuals acquiring the disease over recent years. T2DM complications are mainly caused by vasculopathy and include coronary heart diseases, cerebrovascular diseases, hypertension, and retinal hemorrhage. It can be developed from not only microangiopathy, but also macroangiopathy. Currently, several treatments are available, including insulin, metformin, and insulin sensitizers, such as pioglitazone (PIO) and thiazolidinedione. Apelin is an endogenous peptide that comprises a ligand for the APJ (angiotensin II receptor like-1, AT-1). Both apelin and APJ have been shown to regulate cardiovascular actions such as blood pressure regulation, neovascularization, and cardiac function [1–5]. Both apelin and APJ have also been linked to metabolic activities [6–11], and the serum levels of apelin are usually elevated in both diabetic rats and patients. Several reports have shown that insulin up-regulates the expression of apelin in the adipose tissue [12,13], decreases the serum levels of insulin in diabetic rats, and inhibits the secretion of insulin by the pancreatic β cells in rats [14,15]. Moreover, apelin is involved in glucose homeostasis in insulin-resistant mice [16,17], and Dray et al. have reported that apelin could promote glucose absorption from the intestinal lumen by modifying the ratio of glucose transporters 1 (SGLT-1) and 2 (GLUT2) in the intestine cells of mice [18]. A recent study has reported a significant correlation between single-nucleotide polymorphisms of the apelin gene and diabetes [19]. These observations suggest the key role of apelin in diabetes mellitus not only as a target gene for diabetes drugs but also as a diabetes biomarker. However, another study has reported a lack of association between apelin and insulin resistance, cardiovascular risk factors, and obesity in children [20]. In fact, in different tissues or cells, the expression of apelin/APJ was higher/lower with the same stimulant. For example, Daviaud et al. [21] have reported that TNF-α up-regulated the expression of apelin in the adipose tissue of humans and mice, whereas Melgar-Lesmes et al. [22,23] have revealed that TNF-α decreased the expression of apelin at the mRNA and protein levels in HSCs but increased the expression of apelin in HepG2 cells.

PIO, a peroxisome proliferator activated receptors-γ (PPAR-γ) agonist, is used in clinical practice to treat diabetes as an insulin-sensitizing drug. If used appropriately, it effectively lowers glucose levels in T2DM patients with a low incidence of side effects. Moreover, PIO has the potential cardiovascular benefits that may influence cardiovascular risks, including insulin sensitivity, lipoprotein profile, blood pressure, and inflammation [24]. However, until recently, PIO lacked strong, convincing data supporting its use to prevent vascular-disease-related complications in T2DM patients. Krüppel-like factor 4 (KLF4) is a zinc-finger transcription factor that regulates several cellular biological processes such as cell differentiation, proliferation, apoptosis, and homeostasis maintenance. KLF4 also plays a pivotal role in cell phenotypic switching by regulating the expression of cell proproliferation or prodifferentiation genes and cell-cycle-regulatory genes. Recent studies have also demonstrated that KLF4 activity can be regulated by all-trans retinoic acid (ATRA), transforming growth factor-β (TGF-β), PIO, and other transcription factors [25–27]. Thus, KLF4 can activate or repress gene transcription, depending on the interaction partners and the promoter context [28]. Both PIO and KLF4 play important roles in cardiovascular diseases, and apelin can promote cell proliferation in angiogenesis and vascular remodeling. Our previous study has demonstrated that KLF5 is involved in the regulation mechanism of the expression of apelin [29]. However, whether apelin was regulated by KLF4 or PIO in vascular smooth muscle cells (VSMCs) remains unknown. The present study, aimed to elucidate whether and how KLF4 or PIO regulate the expression of apelin both *in vivo* and *in vitro*.

## Materials and methods

### Animals

Male Sprague–Dawley (SD) rats were purchased by the Institutional Animal Care and Use Committee of the Charles river (Beijing, China, License number: SCXK 2012-0001). The study protocol followed the United States NIH guidelines [Guide for the Care and Use of Laboratory Animals (1985), DHEW Publication NO. (NIH) 85–23: Office of Science and Health Reports, DRR/NIH, Bethesda, MD, U.S.A.] and the ethical standards of Animal Research Ethics Committee of Henan University (The experimental number: HUSOM-2018-310). The rats were bred and housed in a specific pathogen-free environment at the Key Laboratory of Receptors-Mediated Gene Regulation and Drug Discovery of School of Basic Medicine, Henan University. Male SD rats were maintained under standardized conditions and fed with a high-fat and high-sugar diet for 4 weeks, after which diabetes was induced by a single intraperitoneal streptozotocin injection (35 mg/kg, STZ), which was dissolved in a citrate solution (pH = 4.6). Control rats were intraperitoneally injected with the same volume of citrate solution alone. Three days after STZ administration, blood was collected from the tail vein of the rats and analyzed for serum blood glucose (ACCU-CHEK PREFORMA). Rats showing a fasting blood glucose level of >250 mg/dl were considered diabetic and used for the study. Diabetic rats were divided into two groups (6 animals/group): the STZ model group and the STZ + PIO treatment group. The STZ + PIO group received intragastric PIO 20 mg/(kg·d) daily for 6 weeks, whereas the control and STZ groups received a similar dose of intragastric saline during the same period. After 6 weeks, all the rats were anesthetized with intraperitoneal ketamine (60 mg/kg of body weight) and xylazine (5 mg/kg of body weight). Blood was collected through the inner orbital canthus for detection of apelin and insulin and through the thoracoabdominal aorta for qRT-PCR and immunohistochemistry.

### Cells, cell culture, and treatment

Male SD rats (80–100 g) were anesthetized with intraperitoneal ketamine and xylazine, and VSMCs were extracted from the thoracic aorta as described previously [30]. The rats were then killed by exsanguination under general anesthesia. The VSMCs were maintained in Dulbecco’s modified Eagle’s medium (DMEM) supplemented with 10% FBS in a humidified atmosphere with 5% CO_2_ at 37°C. The cells were passaged for three to six generations. Prior to PIO stimulation, VSMCs were maintained in serum-free DMEM for 24 h and then cultured in DMEM supplemented with 2% FBS and 20 μM PIO (Sigma-Aldrich) for the indicated times.

### RNA preparation and quantitative reverse transcription-PCR (qRT-PCR)

Total RNA was isolated with TRIzol® reagent (Invitrogen) according to the manufacturer’s instructions. Glyceraldehyde-3-phosphate dehydrogenase (GAPDH) gene primers were used as internal controls for RNA template normalization. The quantitative PCR of apelin and KLF4 was performed using a Platinum SYBR Green qPCR SuperMix UDG Kit (Invitrogen). The following primers were used: apelin, 5′AGACCCCGGAGGCTAAGGAGTT3′ (sense) and 5 5′TCCGTCATAGTGTCCTCCATCA3′ (antisense); KLF4, 5′CCCCTCTCTCTTCTTCGGACTC3′ (sense) and 5′CCTGGTGGGATAGCGAGTTGGA3′ (antisense); and GAPDH, 5′GCTCTCTGCTCCTCCCTGTT3′ (sense) and 5 5′GTGGCAGTGATGGCATGGAC3′ (antisense). The relative expression levels were calculated using the following equation: relative gene expression = 2^−∆∆CT^.

### Adenovirus expression vector and plasmid constructs

pEGFP-KLF4 and pAd-KLF4 constructs were kindly provided by Dr Wen (the Key Laboratory of Neural and Vascular Biology, China Administration of Education, Hebei Medical University). For the promoter assay, the promoter regions of apelin were amplified by PCR and cloned into the pGL3-Basic vector (Promega) to generate the apelin promoter–reporter construct pGL3-apelin-luc. Luciferase reporter plasmids bearing the rat apelin promoter region were constructed following standard procedures. The primers for apelin were 5′atggGGTACCGTCATCCTCATCCCAGTTAACA3′ (sense) and 5′ctccCCCGGGCCGCGACTCCCAACTACCCGT3′ (antisense).

### Site-directed mutagenesis

Site-directed mutagenesis was performed using a QuikChange ®Site-Directed Mutagenesis kit (Agilent Technologies-Stratagene) according to the manufacturer’s instructions. Various deletion mutants of the apelin promoter–reporter plasmid were verified by sequencing. The primers used to generate a mutation in the putative GKLF site were 5′TGCCTCCCCCCCCCTTGTTCCCGCCTCCTA3′ (sense) and 5′TAGGAGGCGGGAACGGGGGGGGGGGAGGCA3′ (antisense).

### Small interfering RNA (SiRNA transfection)

SiRNAs targeting rat KLF4 (si-KLF4) and nonspecific siRNAs (si-NS) were purchased from Santa Cruz Biotechnology. Transfection was performed using a Lipofectamine reagent (Invitrogen) according to the manufacturer’s instructions. At 24 h post-transfection, VSMCs were treated with or without PIO (20 μM). Cells were then harvested and used for qRT-PCR and Western blotting assays.

### Luciferase assay

Luciferase assays were performed as described previously [31]. VSMCs (3 × 10^4^ cells/well) were seeded into a 24-well plate and grown for 24 h prior to transfection with either reporter plasmids or the control reporter plasmid pRL-TK using a Lipofectamine 2000 reagent (Invitrogen) according to the manufacturer’s instructions. At 24 h post PIO stimulation, the cells were lysed, and luciferase assays were performed using a dual-luciferase assay kit (Promega). Specific promoter activity was expressed as the relative activity ratio of firefly and Renilla luciferases. All the promoter constructs were evaluated in three or more separate wells per experiment.

### Western blotting assays

Crude proteins were extracted from VSMCs as described previously [32], resolved by SDS/PAGE, and transferred onto a PVDF membrane (Millipore). Membranes were blocked with 5% (w/v), nonfat, dried skimmed milk powder in TTBS (100 Mm Tris/HCl, pH 7.5, 150 mM NaCl, and 0.5% Tween 20) for 2 h at 37°C and then incubated overnight at 4°C with the following primary antibodies: 1:300 dilution rabbit anti-apelin (GeneTex), 1:500 dilution rabbit anti-KLF4 (Abcam), and anti-β-actin and rabbit anti-IgG (Santa Cruz Biotechnology) antibodies. After incubation with the appropriate secondary antibody, the immunoreactive signals of antibody–antigens were visualized using the Chemiluminescence Plus Western Blotanalysis kit (Tanon Biotechnology).

### Chromatin immunoprecipitation (ChIP) assay

The ChIP assay was performed according to the manufacturer’s instructions (Millipore). Briefly, VSMCs were treated with 1% formaldehyde for 10 min to cross-link proteins with DNA. The cross-linked chromatin was then prepared and sonicated to an average size of 400–1000 bp. DNA fragments were immunoprecipitated overnight with anti-KLF4 antibodies. After crosslinking reversal, the genomic region of the apelin flanking GKLF site was amplified by PCR with the following primer pairs: 5′CAAGCTGGGAACACAAAACC3′ (sense) and 5′CCTCTGTACTCTCCCCTCAT3′ (antisense).

### Immunohistochemistry

Immunohistochemistry was performed as described previously [33]. Sections were immunostained with anti-apelin or anti-KLF4 antibody (1:100 dilution) and counterstained with hematoxylin. Staining intensities were determined by integrated optical density (IOD) measurement with light microscopy using the computer-based Image-Pro Morphometric System in a double-blind manner.

### Immunofluorescent staining

VSMCs were fixed in 4% paraformaldehyde, permeabilized with 0.1% Triton X-100, incubated with anti-apelin or anti-KLF4 antibody, and further stained with FITC-conjugated secondary antibody. Staining with 4′,6-diamidino-2-phenylindole (DAPI) was used to visualize the nuclear localization. Each section was observed under an inverted fluorescence microscope (Leica).

### Sirius red staining

Sections were deparaffinized and hydrated in distilled water. An adequate picro-sirius red solution was applied to completely cover the tissue section, and the tissue was incubated for 60 min. Subsequently, the slide was quickly rinsed in two changes of acetic acid solution and then rinsed in absolute alcohol, and dehydrated in two changes of absolute alcohol. The slide was cleared and mounted in synthetic resin.

### Statistical analyses

Results are presented as histograms of the means ± SEM for three or more independent experiments. Statistical analyses were performed using either the Student’s *t*-test or one-way ANOVA according to the number of groups compared. A *P* value of < 0.05 was considered statistically significant

## Results

### PIO decreases the serum levels of apelin in T2DM rats

Intraperitoneal STZ injection following HFD led to a significant reduction in the body weights (*P* < 0.05) and a significant increase in the blood glucose level s (*P* < 0.05). The blood glucose levels were significantly lower in the PIO-treated group than those the model group (Table 1). These results indicated that T2DM was successfully established in the rats by the intraperitoneal injection of STZ after HFD and that PIO decreased the blood glucose levels in diabetic rats. Table 2 indicates that the serum levels of apelin in the model group increased more sharply than those in the control group. However, the serum levels of apelin decreased significantly in the rats with PIO gastric perfusion compared with the rats in the model group. To the best of our knowledge, the results reported in the present study showed that for the first time the serum levels of apelin could be reduced by PIO in T2DM rats.

**Table 1:** Blood glucose levels and body weights of the rats before and after pioglitazone treatment

Groups	Con	STZ	STZ+PIO
Body weight (g)			
Initial	160.6 ± 9.56	175.4 ± 5.53	172.2 ± 6.96
Final	478.6 ± 24.62	233.4 ± 13.98*	278.6 ± 8.71
Blood glucose level (mmol/l)			
Initial	6.03 ± 0.31	5.36 ± 0.59	5.63 ± 0.81
Final	6.06 ± 0.35	31.9 ± 0.58*	19.8 ± 1.68^#^

Values are expressed as mean±SEM. *P < 0.05, compared with the Con group, ^#^*P* < 0.05, compared with the STZ group.

**Table 2:** Serum levels of apelin and insulin in rats

Serum level	Groups		
	Con	STZ	STZ+PIO
Apelin (pg/ml)	70.2 ± 14.17	1377.4 ± 340.73*	337.2 ± 90.12^#^
Insulin (ng/ml)	0.19 ± 0.015	0.072 ± 0.006	0.096 ± 0.0087

Values are expressed as mean±SEM. *P < 0.05, compared with the Con group, ^#^*P* < 0.05, compared with the STZ group.

### PIO alleviates collagen fiber deposition in the thoracoabdominal aorta of T2DM rats

**Figure 1:**
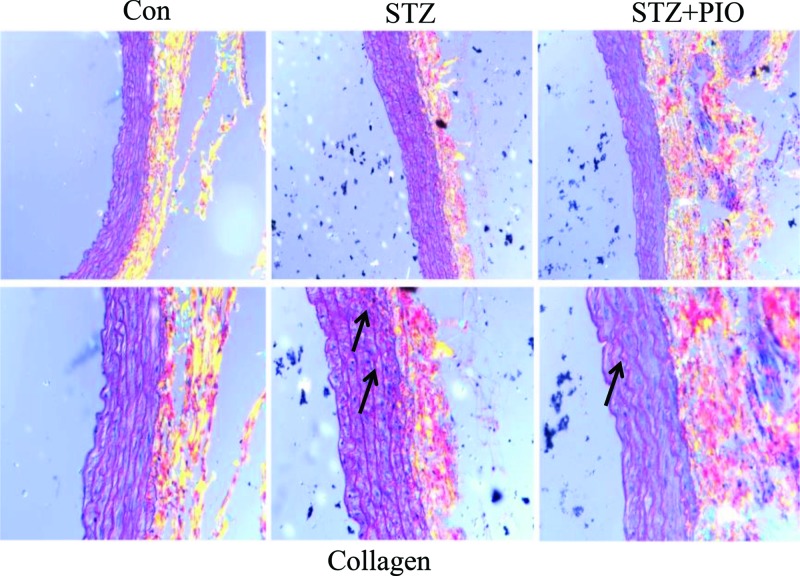
Pioglitazone alleviates collagen fiber deposition in the thoracoabdominal aorta of T2DM rats Sirius red staining of the thoracoabdominal aorta of HFD/STZ rats. Magnification: upper, 200; lower, 400. (*n* = 6 in each group).

Sirius red staining showed that the collagen fiber fragments were deposited in the thoracic abdominal aorta’s media membrane, and the collagen fibers (see arrow) increased more significantly in the rats of the model group than in the rate of the control group. However, the collagen fiber fragments were lower in the aorta of PIO-treated rats than in the aorta of the model rats (Figure 1). These findings suggest that PIO reduces the aortic stiffness in STZ-induced T2DM rats.

### PIO down-regulates the mRNA and protein levels of apelin but up-regulates those of KLF4 in the thoracoabdominal aorta of T2DM rats

**Figure 2:**
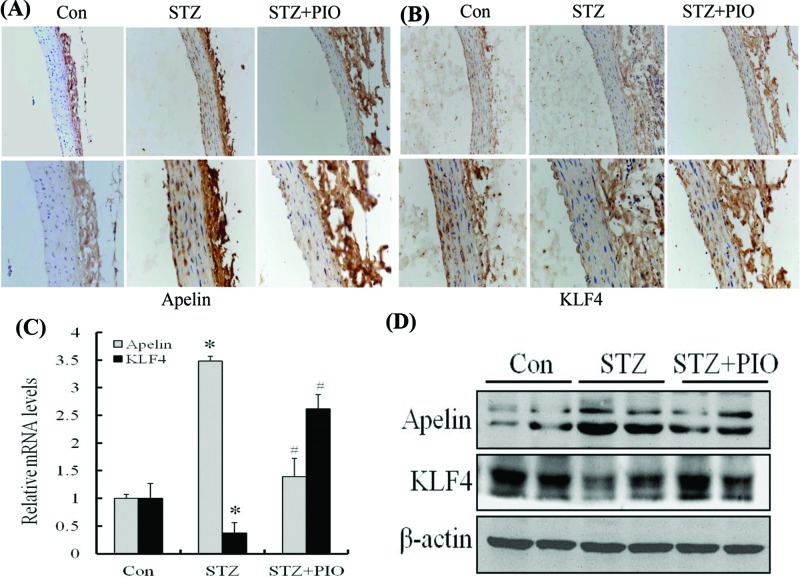
The expression of apelin and KLF4 in the thoracoabdominal aorta of HFD/STZ rats (**A**) Immunostaining of apelin in the aorta of the Con, STZ, and STZ + PIO rats. Magnification: upper, 200; lower, 400. (**B**) Immunostaining of KLF4 in the aorta of Con, STZ, and STZ + PIO rats. Magnification: upper, 200; lower, 400. (**C**) The mRNA levels of apelin and KLF4 were determined by qRT-PCR from the tissues of the thoracoabdominal aorta in rats. (**D**) Crude proteins were extracted from the tissues and then subjected to Western blotting assays with anti-apelin and anti-KLF4. β-actin was used as a loading control. *P < 0.05 compared with the control group. ^#^*P* < 0.05 compared with the STZ group (*n* = 6 in each group).

The expressions of apelin and KLF4 in the thoracic abdominal aorta were detected by immunohistochemistry, qRT-PCR, and Western blotting assays. The results of immunohistochemical staining showed that the aorta of the rats in the model group had a high expression of apelin, whereas the expression of KLF4 was significantly lower. However, PIO decreased the expression of apelin but increased the expression of KLF4 in PIO-treated rats (Figure 2A,B). qRT-PCR analysis showed that the model group exhibited a higher mRNA expression of apelin level and a lower mRNA expression of KLF4 than that of the control group. Therefore, PIO treatment significantly decreased the mRNA expression of apelin in the thoracic abdominal aorta compared with that in the model animals. Instead, the mRNA expression of KLF4 was markedly increased after PIO treatment (Figure 2C). Similar results were obtained by Western blotting assays (Figure 2D), which suggests that the expression of apelin was significantly increased in the thoracic abdominal aorta of the STZ-induced rats, whereas the expression of KLF4 was decreased. Compared with the model rats, PIO increased the expression of KLF4 and decreased the expression of apelin in PIO-treated T2DM rats, suggesting the existence of a relationship between KLF4 and apelin.

### PIO down-regulates the mRNA and protein levels of apelin but up-regulates those of KLF4 in VSMCs

**Figure 3:**
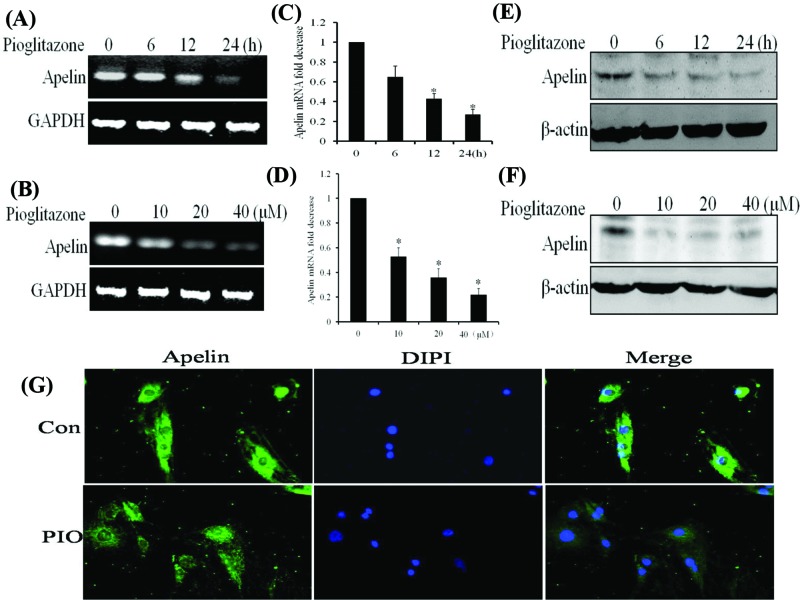
Pioglitazone down-regulates the mRNA and protein levels of apelin in VSMCs All the experiments were performed in triplicate. (**A** and **B**) VSMCs were treated with pioglitazone (20 μΜ) at several time points or at different doses (for 24 h). Total RNA was transcribed with reverse transcriptase and amplified by PCR. GAPDH was used as an internal control. (**C** and **D**) The mRNA levels of apelin were determined by qRT-PCR. The bars represent the mean ± SEM from three independent experiments. *P < 0.05 compared with the respective control group. (**E** and **F**) Western blotting assays were performed using anti-apelin antibodies to examine the expression of apelin at various time points or with different doses. β-Actin was used as a loading control. (**G**) VSMCs were treated with pioglitazone for 24 h and then fixed and stained by immunofluorescence. The expression of apelin in the nucleus and cytoplasm was detected by inverted fluorescence microscopy.

**Figure 4:**
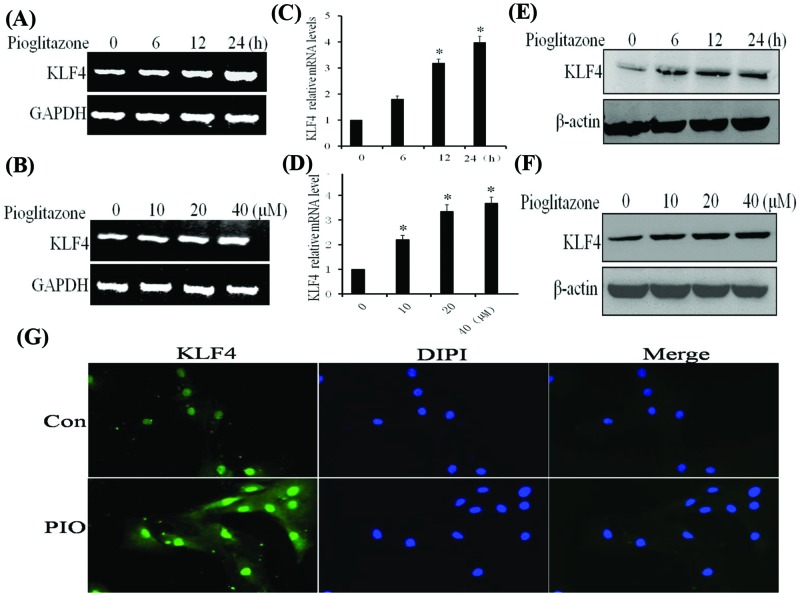
Pioglitazone up-regulates the mRNA and protein levels of KLF4 in VSMCs All the experiments were performed in triplicate. (**A** and **B**) VSMCs were treated with pioglitazone (20 μΜ) at several time points or at different doses (for 24 h). Total RNA was transcribed with reverse transcriptase and amplified by PCR. GAPDH was used as an internal control. (**C** and **D**) KLF4 mRNA levels were determined by qRT-PCR. The bars represent the mean ± SEM from three independent experiments. *P < 0.05 compared with the respective control group. (**E** and **F**) Western blotting assays were performed using anti-KLF4 antibodies to examine the expression of KLF4 at various time points or with different doses. β-Actin was used as a loading control. (**G**) VSMCs were treated with PIO for 24 h and then fixed and stained by immunofluorescence. KLF4 expression in the nucleus was detected by inverted fluorescence microscopy.

Because apelin is closely related to vascular functions and both VSMCs and ESCs can produce apelin, apelin can regulate the biological function in an endocrine and paracrine way. To validate whether the expression levels of apelin were down-regulated in PIO-stimulated VSMCs, the VSMCs were treated with 20 μM PIO several times. The results showed that PIO decreased the mRNA and protein levels of apelin in a time-dependent and dose-dependent manner (Figure 3A–F). Another, Rosiglitazone is principally used for the treatment of diabetes as well as PIO. We have also tested the effect of Rosiglitazone on KLF4 and apelin expression in VSMCs. As shown in Supplementary Figure, Rosiglitazone decreased the protein expression levels of apelin, but increased KLF4 protein levels in VSMC in a time- and dose-dependent manner similarly to PIO. Immunofluorescent staining showed that apelin was located in the cytoplasm of VSMCs. Upon stimulation of the VSMCs with PIO, the basal expression of apelin decreased (Figure 3G). These results are consistent with those of *in vivo* experiments and demonstrate that PIO down-regulates the gene expression of apelin in VSMCs. Moreover, treatment of VSMCs with 20 μM PIO at several time points or at different doses also increased mRNA and protein levels of KLF4 in a time- and dose-dependent manner (Figure 4A–F). Immunofluorescent staining showed that KLF4 was mainly located in the nucleus of VSMCs and that the expression of KLF4 increased in response to PIO signaling (Figure 4G).

### KLF4 negatively regulates the expression of apelin

**Figure 5:**
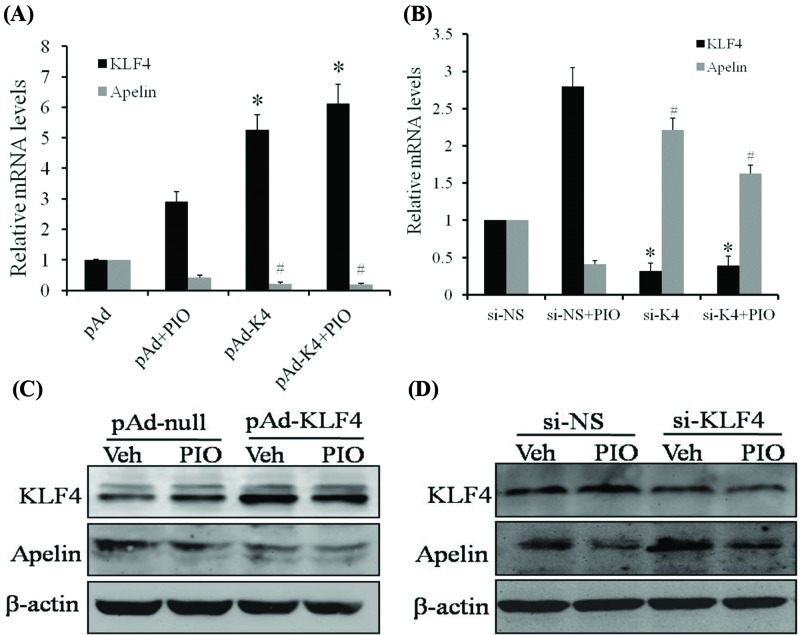
Pioglitazone reduces apelin transcription via KLF4 binding to the GKLF site in the apelin promoter (**A**) Schematic map of the apelin promoter region, showing the position of the GKLF sites. Italic parts represent the mutation base sequence. The arrow represents the primers used for regional amplification in the ChIP assay. (**B**) VSMCs were transfected for 24 h with the apelin promoter–reporter constructs (−2000 bp / -1) or PGL3-basic plasmids and then treated with 20 μM PIO for 24 h. Cell lysates were subjected to luciferase activity assays. Results are presented as relative apelin promoter activity normalized to pRL-TK activity. The bars indicate the mean ± SEM from three independent experiments. *,^#^ P < 0.05 compared with the respective control group. (**C**) VSMCs were co-transfected with the apelin promoter–reporter construct comprising the wild-type GKLF site or GKLF site mutations along with expression plasmids for KLF4 as indicated. Subsequently, they were treated with 20 μM PIO for 24 h, after which, the cell lysates were subjected to a luciferase activity assay. Results are presented as the relative apelin promoter activity normalized to the pRL-TK activity. The bars indicate the mean ± SEM from three independent experiments. (**D**) VSMCs were treated with pioglitazone for 24 h, after which the ChIP assay was performed with antibodies against KLF4. Non-immune IgG was used as a negative control, and the apelin promoter region comprising the GKLF-binding site was amplified by PCR.

To further verify the importance of KLF4 in the expression of apelin, we either infected VSMCs with pAd-KLF4 or transfected them with si-KLF4 to overexpress or knockdown the expression of KLF4, respectively. The qRT-PCR and Western blotting assays results showed that overexpression of KLF4 markedly decreased the basal expression of apelin in terms of transcription and translation levels (Figure 5A,C). Conversely, KLF4 knockdown recovered PIO-decreased the expression of apelin, whereas si-NS did not affect the expression of apelin (Figure 5B,D). These results suggest that KLF4 plays a key role in regulating the expression of apelin by PIO.

### PIO reduces apelin transcription via KLF4 binding to the GKLF site of the apelin promoter

**Figure 6:**
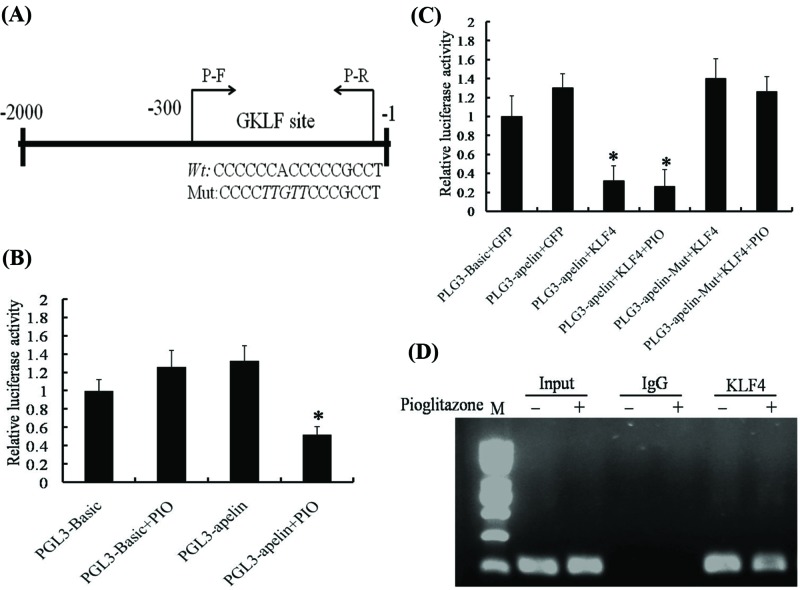
KLF4 negatively regulates the expression of apelin VSMCs were either infected with pAd-null and pAd-KLF4 or transfected with si-NS and si-KLF4 for 24 h and then treated with pioglitazone (20 μM) for 24 h. (**A** and **B**) The total cellular RNA was transcribed with reverse transcriptase and amplified by PCR. GAPDH was used as an internal control to quantify the total amount of cDNA. The bars represent the means ± SEM for three independent experiments. *, *P* < 0.05 compared with the respective control group. (**C** and **D**) Crude proteins extracted from the treated cells were analyzed by Western blotting assays performed with anti-apelin, anti-KLF4, and anti-β-actin antibodies. β-Actin was used as a loading control.

Previous studies have shown the presence of gut-enriched Krüppel-like factor (GKLF) transcription-factor-binding sites in the apelin promoter region and found that the proximal apelin promoter region (−173 to −1 bp) exhibited a greater transcription activity to GKLF [29]. To further investigate the effects of KLF4 and PIO on the apelin promoter activity, VSMCs were cotransfected with GFP–KLF4 expression plasmids and apelin promoter–reporter constructs of approximately 2000-bp fragments. As shown in Figure 6A,B, the addition of PIO decreased the relative activity of the apelin promoter. Overexpression of KLF4 significantly reduced the apelin promoter activity in VSMCs. Similarly, with mutation of the GKLF-binding site, as shown in Figure 6A, the transcription activity of the apelin promoter was restored (Figure 6C). To validate whether KLF4 binds directly to the GKLF-binding site within intact chromatin, a ChIP assay was performed, and the apelin promoter region was analyzed by PCR using primers that selectively amplify the sequence comprising the GKLF binding site. The results showed that the DNA fragments comprising the GKLF-binding site were detected in the immunoprecipitates pulled down by anti-KLF4 and that PIO treatment decreased the binding activity of KLF4 to the GKLF-binding site (Figure 6D). These results confirmed that PIO reduced apelin transcription via KLF4 binding to the GKLF site in the apelin promoter.

## Discussion

Previous studies have shown that the expression of apelin *in vivo* and *in vitro* is upregulated by hypoxia-inducible factor 1-α (HIF1-α), ATRA, and insulin [22,34,35] and down-regulated by TNF-α and the activating transcription factor 4 in different cell types [23,36]. However, the underlying mechanism of the regulation of the expression of apelin by PIO in VSMCs remains unclear. To the best of our knowledge, the present study demonstrated for the first time that PIO down-regulates the gene expression of apelin by promoting KLF4 transcription of the apelin promoter. Previous results have shown that PIO increases the expression of KLF4 by the PPAR-γ binding KLF4 promoter site [37] and confirmed that PIO increased the expression of KLF4 but decreases the expression of apelin both in vivo and in vitro. As a PPAR-γ agonist and insulin-sensitizing drug, PIO is involved in the metabolism of glucose and exerts cardiovascular protective effect in diabetes mellitus. Previous study has shown that PIO could suppress vascular inflammation by increased plasma 15-epi-LXA4 level in patients with T2DM [38]. PIO limited infarct size in myocardial ischemia reperfusion model of rat through inducing the expression and activity of cPLA2 and COX-2 [39]. However, other mechanisms underlying the protection from macroangiopathy by PIO in T2DM remained unknown. Therefore, we established a T2DM rat model, which was induced through the intraperitoneal administration of STZ after consumption of high-fat and high-sugar diets. The present study showed that PIO significantly decreased the blood glucose levels in diabetic rats. Simultaneously, we observed a decrease in the expression of apelin and an increase in the expression of KLF4 in the thoracic abdominal aorta of rats in the PIO-treated group.

One of the main characteristics of macroangiopathy in T2DM is increased aortic stiffness manifested as aortic collagen deposition. Accordingly, the present study showed that the deposition of collagen fiber fragments in the aorta was lower in the PIO-treated rats than in the model rats, indicating that PIO has beneficial effects on the prevention of diabetic macroangiopathy. Previous studies have reported that apelin promotes collagen deposition and increases the expression of MMP-2 or MMP-9 in overnourished mice or rats with hypertension-induced left ventricular hypertrophy [40,41]. It was also found that KLF5 promoted collagen deposition in the hepatic stellate cells of liver fibrosis rat models [42], whereas KLF4 inhibited the synthesis of collagen in human hepatic stellate cells [43]. The present results are consistent with reported in the aforementioned studies. Our previous study reported that KLF5 increases the expression of apelin in a common rat model of carotid artery injury and that the transcription activity of the apelin promoter is increased by the endothelial myocyte enhancer factor 2 (MEF2) transcription factors in a developing cardiovascular system [44]. Were apelin, as a proliferative factor, and KLF4 as an antiproliferative or prodifferentiation transcription factor, related in a regulatory manner? As a zinc-finger-containing nuclear transcription factor, KLF4 might regulate the expressions of several genes. It could either activate or repress gene transcription, thereby regulating cellular biological processes such as cell differentiation, proliferation, development, inflammation, and apoptosis. Previous studies have shown that KLF4 can be activated by several induction factors such as ATRA, platelet-derived growth factor-BB (PDGF-BB), TGF-β, interferon-γ, cyclic adenosine monophosphate (cAMP), and PIO [25,26,45–47].

Our previous study demonstrated the presence of GKLF transcription-factor-binding sites in the apelin promoter region and validated that the proximal apelin promoter region (−173 to −1 bp) exhibited a greater transcription activity than the GKLF-binding site [48]. The luciferase assay identified that overexpression of KLF 4 and PIO treatment significantly decreased the activity of the apelin promoter, whereas the transcription activity was restored when the GKLF-binding site was mutated in VSMCs. The ChIP assay further confirmed the direct binding of KLF4 to the GKLF-binding site within intact chromatins. These results confirm that PIO decreases the expression transcription and translation of apelin through binding of KLF4 to the GKLF-binding site. In the present study, the performed assays showed that apelin was transcriptionally downregulated by the transcription factor KLF4 through PIO treatment. KLF4 was identified as a transcription factor involved in the regulation of the expression of apelin and bound to the GKLF-binding site within the apelin promoter. We believe that this was the first report showing that PIO down-regulates the expression of apelin by promoting KLF4 binding to the GKLF-binding site.

To further verify the importance of KLF4 in regulating the expression of apelin, the following observations were evidenced through experiments where VSMCs were infected with pAd-KLF4 or transfected with si-KLF4. First, overexpression of KLF4 with pAd-KLF4 down-regulated the expression of apelin, while inducing the expression of KLF4. In addition, KLF4 knockdown with si-KLF4 restored the PIO-decreased the expression of apelin. These results are consistent with those of the luciferase assay, in which KLF4 played a key role in regulating the expression of apelin by PIO.

In summary, we showed that PIO signaling down-regulates the expression of apelin by promoting the binding activity of transcriptional factor KLF4 to the apelin promoter. Therefore, the present results describe a novel cell signaling pathway of aorta protection by PIO through the inhibition of the expression of apelin via promoting KLF4 transcription in the apelin promoter. Furthermore, our results may provide a novel therapeutic strategy for macroangiopathy in T2DM, which would help in redefining the biological functions of PIO and KLF4.

## Supplementary Material

Supplementary Figure S1

## Abbreviations

DAPI: 4′,6-diamidino-2-phenylindole
KLF4: Krüppel-like factor 4
PIO: Pioglitazone
PPAR-γ: proliferator activated receptors-γ
T2DM: Type 2 diabetes mellitus
VSMC: vascular smooth muscle cell
