# Supplementary material for: Pioglitazone protects blood vessels through inhibition of the apelin signaling pathway by promoting KLF4 expression in rat models of T2DM
Source: Biosci Rep. 2019 Dec 24;39(12):BSR20190317. doi: 10.1042/BSR20190317 (PMC6928522; doi:10.1042/BSR20190317)
Supplement: Supplementary Figure S1 [file BSR-2019-0317_supp.pdf]

Pioglitazone protects blood vessels through inhibition of the apelin signaling pathway by promoting KLF4 expression in rat models of T2DM

Yin Wang[1,2], Ruonan Zhang[3], Hailin Shen[2], Jing Kong[2], Xinrui Lv[2]*

[1]The First Affiliated Hospital, [2]The Key Laboratory of Receptors-Mediated Gene Regulation and Drug Discovery of School of Basic Medicine, Henan University, Kaifeng, Henan, 475004, China; [3]School of Chinese Integrative Medicine, Hebei Medical University, Shijiazhuang, 050017, China

Co- first author: Yin Wang, Ruonan Zhang

Correspondence: Xinrui Lv (lvxinrui@126.com)

Cells, cell culture, and treatment

Male SD rats (80–100 g) were anesthetized with intraperitoneal ketamine and xylazine, and VSMCs were extracted from the thoracic aorta as described previously (30). The rats were then euthanized by exsanguination under general anesthesia. The VSMCs were maintained in Dulbecco's modified Eagle's medium (DMEM) supplemented with 10% FBS in a humidified atmosphere with 5% CO_2 at 37 °C. The cells were passaged for three to six generations. Prior to Rosiglitazone stimulation, VSMCs were maintained in serum-free DMEM for 24 h and then cultured in DMEM supplemented with 2% FBS and 20 μM Rosiglitazone (Sigma-Aldrich) for the indicated times.

Western blotting assays

Crude proteins were extracted from VSMCs as described previously (32), resolved by SDS/PAGE, and transferred onto a PVDF membrane (Millipore). Membranes were blocked with 5% (w/v), nonfat, dried skimmed milk powder in TTBS (100 Mm Tris/HCl, pH 7.5, 150 mM NaCl, and 0.5% Tween 20) for 2 h at 37°C and then incubated overnight at 4°C with the following primary antibodies: 1:300 dilution rabbit anti-apelin (GeneTex), 1:500 dilution rabbit anti-KLF4 (Abcam), and anti-β-actin (Santa Cruz Biotechnology) antibodies. After

incubation with the appropriate secondary antibody, the immunoreactive signals of antibody–antigens were visualized using the Chemiluminescence Plus Western Blotanalysis kit (Tanon Biotechnology).

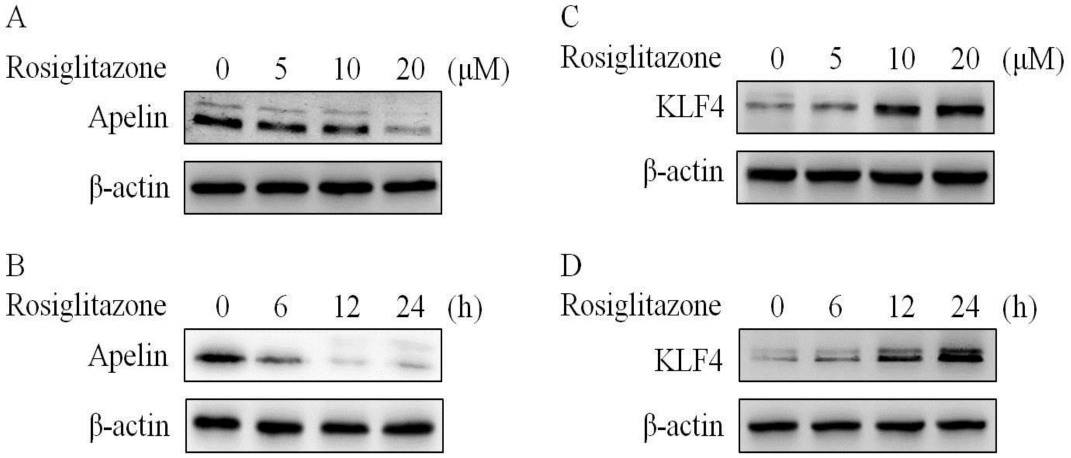

Supplementary Figure. Rosiglitazone down-regulates apelin protein levels but up-regulates KLF4 protein levels in VSMCs. All experiments were performed in triplicate. (A–D) VSMCs were treated with Rosiglitazone at different doses (for 24 h) or at several time points (20 μM) . (A–B) Western blotting was performed using anti-apelin antibodies to examine the expression of apelin. (C–D) Western blotting was performed using anti-KLF4 antibodies to examine the expression of KLF4. β-actin was used as a loading control.
